# Spraying for Diseases of the Upper Air Tract

**Published:** 1906-04

**Authors:** 


### Spraying For Diseases of the Upper Air Tract

Dr. David Walsh, senior physician to the Western Skin Hospital, London (*Medical Press and Circular*) says:

Glycothymoline was brought to my notice as an excellent lotion for nasal and oral sprays and washes. On due inquiry it was found to fulfill the two conditions usually recognized by medical men in the United Kingdom as vouching for the character, so to speak, of such a preparation. First, its advertisements are accepted by our three leading journals, the Lancet, British Medical Journal and the Medical Press and Circular. Second, its composition is not a secret, its formula being freely published. Under these circumstances, I determined to try the effect of this preparation in a few suitable cases. As a general antiseptic fluid that does not coagulate albumin, and is non-irritant, deodorant and practically non-poisonous, glycothymoline has clearly a wide range of usefulness. My own observation, however, has been practically confined to its use in the nose and mouth, with results that have proved satisfactory in every instance, especially in acute coryza, pharyngitis, influenza and septic conditions of the mouth.

